# Extracorporeal carbon dioxide removal for acute hypercapnic exacerbations of chronic obstructive pulmonary disease: study protocol for a randomised controlled trial

**DOI:** 10.1186/s13063-019-3548-4

**Published:** 2019-07-30

**Authors:** Nicholas A. Barrett, Eirini Kostakou, Nicholas Hart, Abdel Douiri, Luigi Camporota

**Affiliations:** 1 Department of Critical Care, Guy’s and St Thomas’ NHS Foundation Trust, Westminster Bridge Rd, London, SE1 7EH UK; 2 Centre for Human & Applied Physiological Sciences (CHAPS), School of Basic & Medical Biosciences, Faculty of Life Sciences & Medicine, King’s College London, London, UK; 3 Lane Fox Respiratory Unit, Guy’s and St Thomas’ NHS Foundation Trust, Westminster Bridge Rd, London, SE1 7EH UK; 4 School of Population Health & Environmental Sciences, King’s College London, London, WC2R 2LS UK; 5 National Institute for Health Research Biomedical Research Centre, Guy’s and St Thomas’ NHS Trust and King’s College London, London, UK

**Keywords:** Acute exacerbations of chronic obstructive pulmonary disease, COPD, Extracorporeal CO_2_ removal, ECCO2R, Non-invasive ventilation, NIV

## Abstract

**Background:**

Chronic obstructive pulmonary disease (COPD) is a common cause of chronic respiratory failure and its course is punctuated by a series of acute exacerbations which commonly lead to hospital admission. Exacerbations are managed through the application of non-invasive ventilation and, when this fails, tracheal intubation and mechanical ventilation. The need for mechanical ventilation significantly increases the risk of death. An alternative therapy, extracorporeal carbon dioxide removal (ECCO_2_R), has been shown to be efficacious in removing carbon dioxide from the blood; however, its impact on respiratory physiology and patient outcomes has not been explored.

**Methods/design:**

A randomised controlled open label trial of patients (12 in each arm) with acute exacerbations of COPD at risk of failing conventional therapy (NIV) randomised to either remaining on NIV or having ECCO_2_R added to NIV with a primary endpoint of time to cessation of NIV. The change in respiratory physiology following the application of ECCO_2_R and/or NIV will be measured using electrical impedance tomography, oesophageal pressure and parasternal electromyography. Additional outcomes, including patient tolerance, outcomes, need for readmission, changes in blood gases and biochemistry and procedural complications, will be measured. Physiological changes will be compared within one patient over time and between the two groups. Healthcare costs in the UK system will also be compared between the two groups.

**Discussion:**

COPD is a common disease and exacerbations are a leading cause of hospital admission in the UK and worldwide, with a sizeable mortality. The management of patients with COPD consumes significant hospital and financial resources. This study seeks to understand the feasibility of a novel approach to the management of patients with acute exacerbations of COPD as well as to understand the underlying physiological changes to explain why the approach does or does not assist this patient cohort. Detailed respiratory physiology has not been previously undertaken using this technique and there are no other randomised controlled trials currently in the literature.

**Trial registration:**

ClinicalTrials.gov, NCT02086084.

**Electronic supplementary material:**

The online version of this article (10.1186/s13063-019-3548-4) contains supplementary material, which is available to authorized users.

## Background

Chronic obstructive pulmonary disease (COPD) is a syndrome characterised by progressive and not fully reversible expiratory airway flow limitation. The underlying pathophysiology of COPD is the progressive destruction of the elastic and alveolar tissue within the lung, resulting in reduced expiratory flow rates, maldistributed ventilation, gas trapping and hyperinflation, which in turn lead to diaphragmatic flattening and inefficient respiration. COPD is punctuated by recurrent exacerbations characterised by a worsening of the patient’s dyspnoea, cough and/or sputum production beyond day-to-day variations [1]. Acute exacerbations are an important cause of hospital admission and impact on patients’ quality of life [2–5], as well as having a huge societal impact amounting to ~ 6% of the total healthcare cost [6]. Exacerbations also accelerate disease progression, leading to a progressive stepwise functional decline [7–13].

Patients with significant exacerbations of COPD resulting in hypercapnic respiratory acidosis (PaCO_2_ > 6 kPa (45 mmHg) and pH < 7.35) have life-threatening respiratory failure and non-invasive ventilation (NIV) has significant mortality benefits for patients [1, 14–19]. NIV provides both inspiratory and expiratory pressure, which, by splinting open collapsing airways by matching intrinsic positive end expiratory pressure (PEEP) and providing positive pressure to assist tiring respiratory muscles, enhances ventilation. This enables an improved ventilation–perfusion relationship, improves gas exchange and simultaneously reduces oxygen consumption and carbon dioxide production because of the reduction in work of the respiratory muscles [16, 20]. Although NIV has substantially improved mortality (risk ratio 0.54, 95% CI 0.38 to 0.76) and decreased the need for intubation (risk ratio 0.36; 95% CI 0.28 to 0.46) in patients with an acute exacerbation of COPD [16], 15–30% of patients commencing NIV fail and require intubation and mechanical ventilation [21–23]. Risk factors for NIV failure include obtundation, hypercapnoea, persisting respiratory acidosis (pH < 7.30), higher APACHE II score (> 28), tachypnoea (> 29) and lack of improvement in the first 1–2 h of therapy [23–27]. Patients who fail NIV and require intubation have a significantly higher mortality than those who improve with NIV (30% vs < 10%) [28], with mortality rates up to 57% in some case series [29].

Extracorporeal CO_2_ removal (ECCO_2_R) is the use of an extracorporeal circuit with a gas-exchanging membrane to provide clearance of CO_2_ directly from the blood [30, 31]. ECCO_2_R has been reported to clear CO_2_ in the pre-clinical setting [32–37] as well as in uncontrolled case series which have included patients with acute exacerbation of COPD (AECOPD) [35, 38–42]. Potential benefits, including avoidance of intubation and early extubation, have been suggested from this retrospective work; however, a retrospective propensity matched case-control study using ECCO_2_R in COPD reported no significant improvement in mortality [43].

In addition to an unclear mortality benefit, it is also uncertain what the impact of ECCO_2_R is on native lung physiology. There is a widely reported reduction in respiratory rate and minute ventilation with improvements in pH and partial pressure of arterial CO_2_ [33, 39, 41, 42, 44, 45]. A small pilot study reported on the changes associated with ECCO_2_R in patients with COPD weaning from a ventilator and found that intrinsic PEEP, inspiratory pulmonary resistance and work of breathing were all reduced [46]. The authors postulated that part of the reduction in arterial CO_2_ seen in these patients was due to both the direct effects of CO_2_ removal and the reduced respiratory muscle work.

Despite ECCO_2_R showing promise as a method to reduce respiratory work and avoid intubation in uncontrolled case series and a propensity analysis, there have been no randomised controlled trials of ECCO_2_R in AECOPD and limited data are available on the physiological impact of ECCO_2_R on respiratory function and work of breathing in AECOPD. Hence, the key clinical role and physiological effect of ECCO_2_R needs greater definition and this work is aimed to explore both the outcome for patients with AECOPD commenced on ECCO_2_R and to better understand the physiological effects of ECCO_2_R in COPD exacerbations.

## Trial hypothesis and objectives

The main hypotheses for the trial are that ECCO_2_R will lead to a significant reduction in the need for NIV and that this is mediated through a reduction in work of breathing secondary to the reduced CO_2_ burden. To explore work of breathing, several methods of assessing work of breathing will be undertaken: oesopheageal pressure monitoring, electrical impedance tomography and parasternal electromyography. These methods have not been used in combination before in this population; however, the benefit of using multimodal monitoring in the setting of acute exacerbations of COPD is that it will allow correlation between the different measurements and it is possible that one or more methods may not be able to be used in an individual patient.

## Methods/design

### Design

The trial is a prospective, randomised, controlled, with 1:1 allocation ratio unblinded study of ECCO_2_R in adults with AECOPD at risk of failing NIV as demonstrated by a persisting pH < 7.30 due to hypercapnoea after initial medical therapy and at least 1 h of NIV. The study will randomise a total of 24 patients, 12 in each treatment arm. Allocation was generated by computer-generated random number and concealed via opaque sealed envelopes.

### Inclusion criteria

- Known COPD with an acute exacerbation. An acute exacerbation is defined as per the GOLD criteria as an increase in dyspnoea, cough and/or sputum over the patient’s normal symptoms. A severe exacerbation is defined as one requiring hospital admission. Patients are known to have COPD when they have a previous diagnosis made and recorded on their medical records, data in support of this will include chest imaging, pulmonary function tests and clinical history/examination.
- Patients with a persistent arterial pH < 7.30 due primarily to hypercapnic respiratory failure after standard medical therapy and at least 1 h of NIV.
- Age over 18

### Exclusion criteria

- Haemodynamic instability after ensuring euvolaemia
- Multiple organ failure requiring other organ supportive therapy, including indication for intubation and mechanical ventilation; this includes requirement for chronic organ supportive therapy (e.g. chronic non-invasive ventilation, dialysis)
- Known allergy/intolerance of heparin including known heparin-induced thrombosis and thrombocytopaenia
- Acute uncontrolled haemorrhage
- Intracerebral haemorrhage
- Recent (< 6 months) ischaemic cerebrovascular accident
- Organ transplant recipient
- Expected to die within 24 h or with limitations of therapy in place precluding admission to critical care
- Venous abnormality or body habitus precluding cannulation
- Contraindication to NIV (as per British Thoracic Society recommendations):
- Facial burns/trauma/recent facial or upper airway surgery
- Vomiting
- Fixed upper airway obstruction
- Undrained pneumothorax
- Recent upper gastrointestinal surgery
- Inability to protect the airway
- Life-threatening hypoxaemia (PaO_2_/FiO_2_ < 20 kPa)
- Bowel obstruction
- Patient refusal
- Pregnancy
- Severe hepatic failure (ascites, hepatic encephalopathy or bilirubin > 100 μmol/L)
- Severe chronic cardiac failure (NYHA class III or IV)
- Bleeding diathesis (INR > 1.5, platelets < 80,000) in the absence of anticoagulation therapy

### Primary outcome

The primary outcome is the time taken to discontinuation of NIV in patients with acute, severe exacerbations of COPD through the addition of ECCO_2_R to standard care. The definition of discontinuation of NIV is cessation of NIV for at least 6 h. Time to discontinuation will be measured from randomisation. The reason for discontinuation will be collected and successful discontinuation consists of being free of mechanical ventilation.

### Secondary outcome

Secondary outcomes include the impact of ECCO2R on:

- Safety, including:
- Incidence of haemorrhagic complications—significant haemorrhage is defined as bleeding requiring transfusion of more than one unit of blood, intracerebral bleeding, bleeding requiring procedural intervention
- Incidence of thrombosis—all patients will have screening duplex ultrasound of the cannulated vessel
- Incidence of haemolysis defined by rising lactate dehydrogenase, bilirubin red cell fragments on a blood film and/or a rising plasma free-haemoglobin (> 0.5 g/dL)
- Complications relating to cannulation including those at insertion—bleeding, vascular damage, pneumothorax and line infection, both clinically apparent (erythema/cellulitis at insertion site) and microbiologically proven (positive blood culture with concomitant line tip culture or line insertion site culture)
- Physiology, including respiratory rate, heart rate, blood pressure, temperature, arterial blood gases measured at time (T)0 min, T30 minutes, T 60 min, then every fourth hour for the first 24 h and then every sixth hour until discontinuation of the acute phase of the trial
- Daily spirometry including forced vital capacity, forced expiratory volume in 1 s and the ratio between them
- Work of breathing, including electrical impedance tomography, parasternal electromyography and oesophageal pressure (these are described in detail below)
- Patient outcomes:
  - Mortality (ICU, hospital and 3 month)
  - Length of stay (ICU, hospital)
  - Need for intubation and where this occurs, duration of mechanical ventilation and need for tracheostomy, NIV failure and need for intubation will be decided by the clinical team managing the patient. Reason for NIV failure will be recorded by the research team.
  - 3-Month hospital readmission rate
  - Tolerance, assessed using both a numerical rating scale (0–10) and visual acuity scale (0–10 cm) for both dyspnoea and comfort with the lowest reading being extremely uncomfortable and the highest being extremely comfortable. Comparisons between the two different measures will be used without an absolute definition of comfort/discomfort.
  - At 3-months following randomisation: COPD assessment test, St George’s Respiratory Questionnaire, EuroQoL 5D. Comparisons between the different measures will be used without an absolute definition of success at follow-up.
- Cost-effectiveness with ICU bed day and consumables cost/quality adjusted life year (QALY)

## Trial procedures

### Recruitment

Potential study participants will be identified by the critical care outreach team, who are required to review every patient placed on NIV either on the wards or in the emergency department. The outreach team will inform the research team of potential study participants. The research team is available 24 h, 7 days.

### Informed consent

Informed consent/assent will be taken by appropriately trained investigators. Informed consent will be obtained prior to any study-related procedures being undertaken. The study participant or, if they lack capacity at the time of enrolment, the patient’s consultee must personally sign and date the latest approved version of the informed consent form before any study-specific procedures are performed.

Written and verbal versions of the participant information and informed consent will be presented to the participants detailing no less than: the exact nature of the study; the implications and constraints of the protocol; the known side effects and any risks involved in taking part. It will be clearly stated that the participant is free to withdraw from the study at any time for any reason without prejudice to future care, and with no obligation to give the reason for withdrawal.

The participant or their consultee will be allowed up to 2 h to consider the information, and the opportunity to question the investigator or other independent parties to decide whether they will participate in the study. Written informed consent will then be obtained by means of participant or their consultee dated signature and dated signature of the person who presented and obtained the informed consent. The person who obtained the consent must be suitably qualified and experienced and have been authorised to do so by the Chief/Principal Investigator. A copy of the signed informed consent will be given to the participants or their consultee. The original signed form will be retained at the study site. Once any participant has regained capacity they will be given written and verbal information about the study. Written informed consent will then be obtained by means of participant dated signature and dated signature of the person who presented and obtained the informed consent. It will be clearly stated that the participant is free to withdraw from the study at any time for any reason without prejudice to future care, and with no obligation to give the reason for withdrawal (Additional files 1).

### Randomisation and codebreaking

Randomisation will be achieved using a computer-generated random list then stored as sequential sealed envelopes accessible to the primary study team. Other sites will randomise by telephone to the lead centre with envelopes opened and accessible only to the primary study team. The study participants, care providers and outcome assessors will be unblinded (open label).

### Data collection

**Fig. 1:**
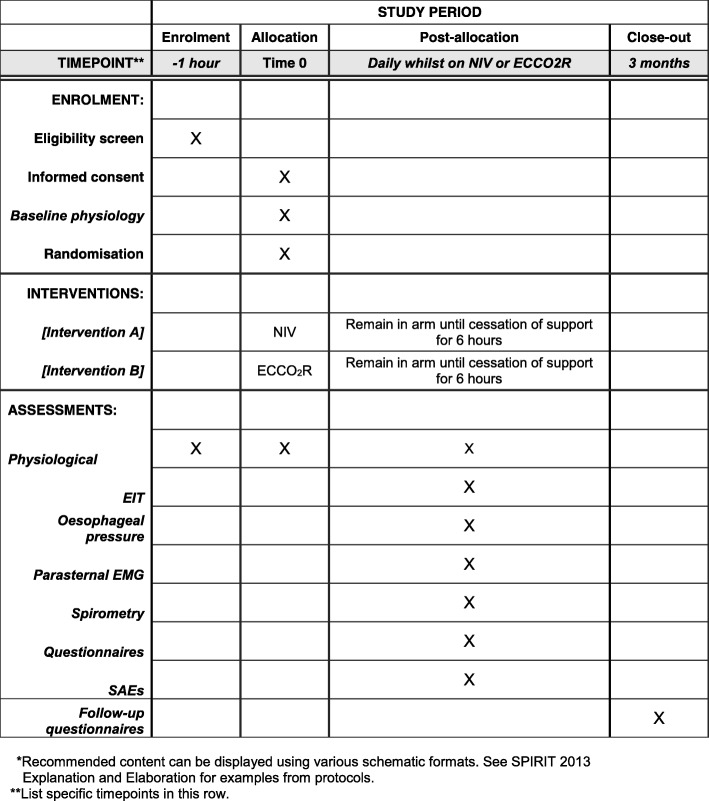
Example template of recommended content for the schedule of enrolment, interventions, and assessments (recommended content can be displayed using various schematic formats; see SPIRIT 2013 Explanation and Elaboration for examples from protocols) (Additional File 2). **List specific timepoints in this row

For eligible patients, clinical details will be collected after consent has been obtained and randomisation undertaken (Fig. 1). This will include details to confirm eligibility and basic demographic and medical details, including acute and chronic co-morbidity and medicines as well as a full physical examination. Details of baseline routine clinical investigations will be recorded. Participants will be allocated a sequential trial number to ensure participant anonymity and confidentiality. All data will be stored in de-identified password-protected databases held within the NHS. All paper documents will be transferred to the department of Critical Care at Guy’s and St Thomas’ NHS Foundation trust and stored in locked, fire-proof cabinets. All data transfers between institutions will conform to the information governance requirement of the NHS.

### Treatment of trial participants

Following presentation, initial medical therapy and NIV will be commenced as per current local and UK national guidelines for the management of AECOPD [47]. All patients with AECOPD and pH < 7.30 after a minimum of 1 h NIV will be eligible for inclusion unless exclusion criteria are met. Once informed written consent is obtained from either the patient or their consultee if consent from the patient is not possible, patients will be randomised to the continuation of NIV alone or addition of ECCO_2_R to NIV. All other aspects of care, including acute, on-going and post-trial care, will be at the discretion of the clinical team. Monitoring will be the standard of care of ICU, including arterial and central venous access where this is deemed appropriate by the clinical team.

Patients who are randomised to continuation of NIV alone will be managed in accordance with hospital protocols and national guidelines for NIV. NIV will be titrated to achieve an arterial pH > 7.35, respiratory rate < 25 and patient comfort with breathing. Discontinuation of NIV will occur when judged appropriate by the patient’s clinician. Cessation of NIV will be defined as occurring after six continuous hours without NIV.

#### ECCO_2_R

Patients who are randomised to addition of ECCO_2_R will undergo cannulation in either the right internal jugular vein or one of the femoral veins using a 15.5 French double lumen ECCO_2_R cannula (patient and operator preference) using an ultrasound-guided Seldinger technique. The ECCO_2_R device, will be primed with 0.9% saline and 1 unit/mL heparin in accordance with the manufacturer’s instructions. The blood flow will be titrated to the maximum achievable (usually 400–500 mL/minute) and sweep gas titrated up to 10 L/minute in 1 L/minute increments every 15 min. ECCO_2_R will be titrated to achieve an arterial pH > 7.35, respiratory rate < 25 and patient comfort with breathing. Discontinuation of NIV and ECCO_2_R will occur when judged appropriate by the patient’s clinician. Cessation of ECCO_2_R will be defined as occurring after six continuous hours without ECCO_2_R. All patients receiving ECCO2R will receive a continuous infusion of heparin to maintain an APTTr of 1.5–2 titrated using the hospital protocol. Data relating to the ICU inpatient stay will be collected from the ICU and hospital information systems. At 3 months following randomisation, a telephone interview will be conducted by the randomising study centre to ascertain the post-discharge survey data.

Whilst the patient remains on respiratory support (either NIV or ECCO_2_R), all patients will undergo daily assessment of dyspnoea and comfort, spirometry, electrical impedance tomography, parasternal electromyography and oesophageal pressure monitoring.

#### Electrical impedance tomography

Electrical impedance tomography (EIT) is a non-invasive, bedside monitoring technique that provides semi-continuous, real-time information about the regional distribution of the changes in electrical resistivity of the lung tissue due to variations in ventilation (or blood perfusion) in relation to a reference state [48–50]. To generate an image, EIT repeatedly injects small alternating electric currents (usually 5 mA) at high frequency of 50–80 kHz through a system of 16 skin electrodes applied circumferentially around the thorax in a single plane between the fourth and sixth intercostal space [51, 52]. While an adjacent pair of electrodes ‘injects’ the current, the remaining passive electrode pairs measure the differences in electric potential. A resistivity (impedance) image is reconstructed from these data by a mathematical algorithm [48–50]. Lungs have a changing impedance during the respiratory cycle due to changes in the air–tissue ratio. This variation in tidal impedance provides quantitative data on both changes in lung volume and regional distribution of air within the lung, allowing the recording of physiological phenomena with high temporal and functional resolution [53–56].

COPD is known to have significant spatial and temporal heterogeneity of ventilation due to the inhomogenous destruction of lung elastic tissue leading to loss of elastic recoil, regional ventilation inequality, increased airway resistance and dynamic hyperinflation. One of the key problems in exacerbations of COPD is the prolongation of the expiratory phase in COPD, resulting in the development of dynamic hyperinflation which leads to haemodynamic compromise, increased work of breathing and ventilator asynchrony [57, 58]. Information derived from EIT indices, including the global inhomogeneity index, can be used to demonstrate both inhomogeneous ventilation and the development of dynamic hyperinflation [59–64]. One potential method to demonstrate dynamic hyperinflation is the mapping of expiratory time constants using the change in impedance over time [65, 66]. It is anticipated that time constants will correlate with other measures of global inhomogeneity and this will be used to describe the physiological consequences of NIV and ECCO_2_R.

EIT will be undertaken unless there is a contraindication (pacemaker or implantable defibrillator). EIT examinations will be performed using the Drager Pulmovista 500 EIT device (Pulmovista®; Draeger, Luebek, Germany) using a silicone band consisting of 16 integrated electrodes and one reference electrode applied around the thorax at the fourth intercostal space in accordance with the manufacturer’s instructions. Real-time analogue waveform outputs from the ventilators feed to an inbuilt data acquisition system (Draeger EIT Data Analysis Tool 6.1). Data will be analysed after processing using custom-made software and in accordance with the method described by the Translational EIT Development (TREND) study group [51]. Data will be displayed to demonstrate changes in tidal volume, end-expiratory lung volume, spatial distribution of gas and respiratory time constants [67–70]. Descriptors of the spatial distribution of ventilation, including the global inhomogeneity index [71–74] and the coefficient of variation [61, 72, 75], will be reported. Additionally, the heterogeneity of ventilation will be quantified measuring regional respiratory time constants [69, 70], phase shifts in regional ventilation [76], the ventilation delay index [77] and heterogeneity of expiratory times [61, 62, 78].

#### Parasternal electromyography

Exacerbations of COPD result in an increase in the subjective work of breathing, anxiety and tachypnoea, which in turn result in a progressive increase in the elevated neural respiratory drive mediated through the respiratory centre in the brainstem [79–81]. Although the output of the respiratory centre cannot be measured directly, a well validated surrogate measure is the electromyography (EMG) signal measured at the level of the parasternal intercostal muscles using surface electrodes placed over the second intercostal space [80, 82–84]. Parasternal EMG has been demonstrated to be a reproducible and well tolerated technique to assess neural respiratory drive [85], particularly for patients with an exacerbation of COPD [80, 81]. To allow physiologically meaningful comparisons of the EMG signal, the root mean square is used to quantify the intensity and duration of the contraction and has been shown to be linearly associated with increasing load on the muscle [86, 87]. Additional measures to quantify the neural respiratory drive are the percentage of the EMG signal of the maximum EMG signal obtained (EMG_para%max_) and the neural respiratory drive index (NRDI), which is the product of EMG_para%max_ and the respiratory rate [80]. NRDI is a validated measure of work of breathing during an acute exacerbation and allows changes in work of breathing over time to be documented [80, 81].

EMG will be recorded from the parasternal second intercostal space bilaterally and then processed and analysed in accordance with previously published work [80, 81]. The root mean square will be used to calculate the root mean square EMG_para%max_ from the maximum EMG signal obtained and the NRDI will be calculated by multiplying EMG_para%max_ by the respiratory rate. Changes in an individual patient will then be analysed over time.

#### Oesophageal pressure monitoring

Oesophageal pressure (P_ES_) may be measured using an inflated balloon placed in the lower oesophagus and attached to an air-filled pressure transducer. The changes in P_ES_ with respiration are concordant with the changes in pleural pressure (P_PL_) [88–90]. The difference between P_ES_ and airway pressure (P_AW_) is a reasonable estimate of transpulmonary pressure (P_L_) in the region surrounding the balloon [89–91]. From this, the work of breathing can be calculated using the integration of the difference between the static compliance of the chest wall and the oesophageal pressure multiplied by volume over time. As chest wall compliance cannot be directly measured in spontaneously breathing patients, it will be estimated as normal, at 200 mL/cmH_2_O [88, 91]. Respiratory muscle work will also be estimated using the pressure time product of the oesophageal pressure (PTP_ES_) [88, 91]. PTP_ES_ is the integral of pressure over time and is the product of the change in pressure multiplied by the duration of the contraction [88, 91]. The particular advantage of PTP_ES_ is that it provides an estimate of activity regardless of whether or not a volume is generated. Due to the development of dynamic hyperinflation in exacerbations of COPD, the muscles of respiration commence contracting prior to airflow commencing [89, 92]. PTP_ES_ estimates of the work undertaken during this isometric phase of contraction. Application of extrinsic PEEP can be titrated to minimise work of breathing [91].

Oesophageal pressure measurements will be undertaken using a 5 French latex free balloon catheter (Cooper Surgical, Conneticut, USA) inserted into the lower oesophagus trans-nasally with 0.5–1.5 mL of air instilled to allow optimal transmission of the pressure waveform [91]. The catheter is attached to a pressure transducer to obtain pressure measurements and simultaneous pneumotachograph recordings are made (Hans Rudolph differential pneumotachograph). The balloon position will be validated using the dynamic occlusion test in the spontaneously breathing patient [92, 93].). All signals will be collected on a personal computer through a 12-bit analogue-to-digital converter (National Instrument DAQCard 700; Austin, TX) at a sampling frequency of 200 Hz (ICU-lab, KleisTEK Engineering, Bari, Italy). Signals will be analysed using ICU-Lab software. The oesophageal pressure trace will be used to calculate work of breathing and the pressure time product will be calculated. Oesophageal pressure will be used solely as an outcome measure and will not be available for use at the bedside.

## Safety reporting

The safety reporting window for this trial is from enrolment until completion of the interventional component of the trial (cessation of the device). Safety reporting will follow the standard operating procedure for Guy’s and St Thomas’ NHS Foundation Trust. Anticipated adverse events will be recorded as part of the trial information safety endpoints. All serious adverse events will be reported to the sponsors and manufacturer within one working day.

## Sample size

It is estimated that the effect of addition of ECCO_2_R will be a reduction in NIV duration from 48 h to 36 h. Group sample sizes of 24 (12 in each arm) patients achieve 80% power to reject the null hypothesis of equal means when the population mean difference is 12 h with a common standard deviation of 10 h with alpha level of 5%.

## Sites and site monitoring

The study will be conducted at up to five sites within the UK and monitored according to the standard operating procedure for site monitoring of the GSTT Trials Unit, which includes at least one site visit over the course of the trial as well as triggered visits if required. All data from the sites will be shared solely with the co-ordinating centre. Local sites will liaise directly with the manufacturers for equipment/disposables. The lead site will supervise all other aspects of the study and is available 24 h, 7 days for questions relating to the study. The randomisation sequence is solely held at the lead centre who will also allocate trial participant numbers. All sites are University-affiliated hospitals with tertiary intensive care units experienced in the provision of extracorporeal CO_2_ removal. Current sites include Guy’s and St Thomas’ NHS Foundation Trust, Manchester University NHS Foundation Trust, Chelsea and Westminster NHS Foundation Trust and Queen Elizabeth University Hospital, Glasgow. Additional data about sites can be obtained from the corresponding author. Source data verification conducted over the course of the trial will be at least 1% and will increase if significant discrepancies are found.

## Protocol amendments

Protocol amendments will be approved by both the sponsor and the REC prior to being implemented. Any protocol amendments will be numbered and dated and shared with investigating centres to ensure version control.

## Data and data monitoring

The study will be conducted in accordance with the current approved protocol, ICH GCP, relevant regulations and standard operating procedures. Audits of data are not planned.

Regular monitoring will be performed according to ICH GCP. Data will be evaluated for compliance with the protocol and accuracy in relation to source documents. Following written standard operating procedures, the monitors will verify that the clinical trial is conducted and data are generated, documented and reported in compliance with the protocol, GCP and the applicable regulatory requirements.

Data will be stored in the department of Critical Care, Guy’s and St Thomas’ NHS Foundation Trust.

## Access to data

Direct access will be granted to authorised representatives from the sponsor, host institution and regulatory authorities to permit trial-related monitoring, audits and inspections. The funder has access only to anonymised information embedded within the device which is not accessible to researchers. All other data remain the property of the researchers.

## Statistical analysis

Summary measures for the baseline characteristics of each group will be presented as mean and standard deviation for continuous (approximate) normally distributed variables, medians and interquartile ranges for non-normally distributed variables, and frequencies and percentages for categorical variables. The planned primary efficacy analyses, difference between the two arms in reduction in NIV duration, will be analysed on randomized patients using a non-parametric approach, Mann-Whitney U test. Similar analyses as for the primary outcome measure will be conducted for secondary efficacy data. Missing data in the efficacy population will not be interpolated. All randomised participants in both arms with valid non-invasive ventilation data will be included in the analysis. Analysis of the physiological data will be per protocol, whilst analysis of outcomes will be intention to treat. Physiological data will be compared within individuals to describe a relative change in work of breathing. The statistical analysis plan can be accessed through the corresponding author. No subgroups have been planned.

## Discussion

The use of ECCO_2_R for AECOPD [94] has progressively increased despite the lack of supporting evidence. This trial is in response to the recent call by NICE to increase the depth of evidence for ECCO_2_R [95]. In keeping with this, the key aims of the trial are to provide outcome, safety and physiological data about the use of ECCO2R in exacerbations of COPD. Given the relatively small size of the study, it is not expected that this work will provide a definitive answer as to the impact of ECCO_2_R on the mortality and intubation/mechanical ventilation in exacerbations of COPD. However, the detailed physiology which will be measured should allow an improved understanding of how ECCO_2_R impacts respiratory physiology. Exacerbations of COPD are one of the leading causes of hospital admission in the UK and the intensive care and hospital costs for patients who do not respond to first-line non-invasive ventilation are significant for the NHS. Consequently, it is important to also measure the economic cost of this approach for patients with significant exacerbations of COPD. The clinical relevance of this work is potentially highly significant—both the mechanisms and outcomes of ECCO2R in AECOPD are the subject of research efforts and clinical interest.

## Publication policy

The trial will be submitted to relevant professional congresses in abstract format as well as submitted to the peer-reviewed literature. Participants will be notified of the trial results in writing, if they consent to be contacted. There are no restrictions on publication.

## Data monitoring committee

Given the number of participants and predominantly physiological endpoints, a data monitoring committee was not required.

## Trial status

At the time of manuscript submission, the trial is still currently recruiting participants.

## Sponsorship

The trial is sponsored by Guy’s and St Thomas’ NHS Foundation Trust, Westminster Bridge Rd, London SE1 7EH, UK. The sponsor carries insurance to compensate any patients who suffer trial-related harm. The sponsor is independent of the design, conduct, analysis and reporting of the study.

## Additional files

Additional file 1: Contains a consultee assent form, Consultee information sheet, the participant consent and the participant information sheet. (ZIP 339 kb)

Additional file 2: SPIRIT 2013 Checklist: Recommended items to address in a clinical trial protocol and related documents*. (DOC 124 kb)

## Data Availability

Data sharing is not applicable to this article as no datasets were generated or analysed during the current study.

## Abbreviations

AECOPD: Acute exacerbation of chronic obstructive respiratory disease
APACHE II: Acute Physiology And Chronic Health Evaluation II
APTTr: Activated partial thromboplastin time ratio
CO_2_: Carbon dioxide
COPD: Chronic obstructive pulmonary disease
ECCO_2_R: Extracorporeal carbon dioxide removal
EIT: Electrical impedance tomography
EMG: Electromyography
EMG_para%max_: Percentage of the electromyography signal of the maximum electromyography signal obtained
FiO_2_: Fraction of inspired oxygen
GOLD: Global Initiative for Chronic Obstructive Lung Disease
ICH GCP: International Committee for Harmonisation of Good Clinical Practice
ICU: Intensive care unit
INR: International normalised ratio
NIV: Non-invasive
NOK: Next of kin
NRDI: Neural respiratory drive index
NYHA: New York Heart Association
PaCO_2_: Arterial partial pressure of carbon dioxide
PaO_2_ /FiO_2_: Arterial partial pressure of oxygen to fraction of inspired oxygen ratio
P_AW_: Airway pressure
PEEP: Positive end expiratory pressure
P_ES_: Oesophageal
P_L_: Transpulmonary pressure
P_PL_: Pleural pressure
PTP_ES_: Pressure time product of the oesophageal pressure
QALY: Quality adjusted life year
REC: Research ethics committee
T: Time
