# Supplementary material for: Extracorporeal carbon dioxide removal for acute hypercapnic exacerbations of chronic obstructive pulmonary disease: study protocol for a randomised controlled trial
Source: Trials. 2019 Jul 30;20:465. doi: 10.1186/s13063-019-3548-4 (PMC6664508; doi:10.1186/s13063-019-3548-4)
Supplement: Supplementary file 1 — Contains a consultee assent form, Consultee information sheet, the participant consent and the participant information sheet. (ZIP 339 kb) [file 13063_2019_3548_MOESM1_ESM.zip › Consultee Assent formR1.docx]

Participant Study Number:

**CONSULTEE DECLARATION FORM**

**Project title: Extra-corporeal CO_2_ Removal as an adjunct to Non-Invasive Ventilation in Acute Severe Exacerbations of COPD**

**Ethics Reference:** 14/EE/0109

**Research & Development Reference:**

**Name of Principle Investigator:** Dr Nicholas Barrett

**Please initial box**

| I _______________________ have been consulted about __________________________ participation in this research project. I confirm that I have read and understood the Consultee Information Sheet (V4.1, dated 16^th^ January 2016) for the above study and have had the opportunity to ask questions about the study and understand what is involved. | ⬜ |
| --- | --- |
| I understand that my relative’s participation is voluntary and that I am free to withdraw him/her at any time, without giving any reason, and without their medical care or legal rights being affected. | ⬜ |
| I understand that relevant sections of my friend's/relative's notes and data collected during the study may be looked at by responsible individuals from Guy’s & St Thomas’ NHS Foundation Trust or from regulatory authorities, where appropriate and relevant to my friend’s/relative’s taking part in the above research. | ⬜ |
| In my opinion, he/she would have no objection to his/her GP being informed about his/her enrolment in the above study | ⬜ |
| In my opinion, he/she would have no objection to taking part in the above study | ⬜ |

­­­­­­­­­­­­­­­­­_________________________

Name of Participant

________________________ ________________ ____________________

Name of Consultee Date Signature

________________________

Consultee’s Relationship to Participant

_________________________ ________________ ____________________

Name of Study Researcher Date Signature

When completed: 1 (original) to be kept in care record, 1 for consultee; 1 for researcher site file
