# Supplementary material for: Extracorporeal carbon dioxide removal for acute hypercapnic exacerbations of chronic obstructive pulmonary disease: study protocol for a randomised controlled trial
Source: Trials. 2019 Jul 30;20:465. doi: 10.1186/s13063-019-3548-4 (PMC6664508; doi:10.1186/s13063-019-3548-4)
Supplement: Supplementary file 1 — Contains a consultee assent form, Consultee information sheet, the participant consent and the participant information sheet. (ZIP 339 kb) [file 13063_2019_3548_MOESM1_ESM.zip › Consultee information sheetR1.docx]

**Consultee Information Sheet**

**Principal Investigator:** Dr Nicholas Barrett

**Location/Study Site:** St Thomas’ Hospital, London, SE1 7EH

**Sponsor:** Guy’s & St. Thomas’ NHS Foundation Trust.

**Participant Study Number:**

**Extra-corporeal CO_2_ Removal as an adjunct to Non-Invasive Ventilation
in Acute Severe Exacerbations of COPD**

**Ethics Reference:** 14/EE/0109

**Research & Development Reference:**

**Introduction:**

We are undertaking a research study involving people with severe acute emphysema or chronic obstructive pulmonary disease (COPD). We are asking you about the participation of a person who is not able to consent for themselves, because he/she lacks the legal capacity to do so. To help decide if he/she should join the study, we would like to ask your opinion whether or not he/she would want to be involved.

Before you decide it is important for you to understand why the research is being done and what it would involve for the participant. Please take time to read this information carefully. One of our team will go through the information with you. Please ask us if there is anything that is not clear or if you would like more information and time to decide. Your decision is completely voluntary. You may discuss it with others if you wish. The decision you make will not affect the participant's care or treatment in any way.

If you decide that he/she would wish to take part in our research study, you will be asked to read and sign an assent form. You will then be given a copy to keep. When the person regains capacity to they will have the study explained to them and they will be asked to consent for themselves.

If you do not think that he/she would wish to participate in our research study or either they or you wish to withdraw from the study after consenting, please say so, as their participation is entirely voluntary. This will not affect the standard of clinical care that they receive.

**Part 1** tells you the purpose of the study and what will happen to him/her if he/she takes part.

**Part 2** gives you more detailed information about the conduct of the study.

Please ask the study researcher if there is anything that is not clear or if you would like more information. Take time to decide whether or not you wish your relative to take part.

**PART 1:**

**1. What is the purpose and background of the study?**

Patients with long-standing chronic obstructive pulmonary disease (COPD) or emphysema commonly have acute exacerbations of their disease causing increased breathlessness, cough and phlegm (or sputum). When exacerbations are mild, they are treated with medications prescribed by GPs and the patient can usually stay at home. More severe exacerbations usually require hospital admission. When exacerbations are very severe and breathing is very difficult, then the carbon dioxide (CO2) levels in the blood can rise. If the CO2 level becomes high enough, the acid levels in the blood rise and the patient can become progressively distressed. If a patient is this ill, the usual treatment often includes prednisolone (a steroid tablet), nebulisers and antibiotics. If the CO2 or acid levels in the blood remain high then patients are usually started on a breathing machine called non-invasive ventilation (NIV), ), which improves breathing by giving extra pressure to reduce the effort of each breath. Research studies in the UK and overseas have previously shown that using NIV for exacerbations of COPD can improve survival rate. NIV is given through a mask fitted tightly onto the patient’s face. The mask needs to be tight to prevent air-leak and facilitate the correct functioning of the breathing machine. Patients who are started on NIV have a good chance of surviving to leave hospital (approximately 14 out of 15 patients). However some patients, approximately 1 in 4 or 5, fail NIV. Some patients fail NIV because they find it uncomfortable, whilst other patients get sicker despite NIV. If a patient fails NIV, usually the next level of treatment is for the doctors to insert a breathing tube through the mouth down into the trachea (the large airway leading air from the mouth to the lungs) under deep sedation. and to start on a different kind of breathing machine called a mechanical ventilator. Approximately 1 in 3 patients with COPD who are started on a mechanical ventilator do not survive, because of increased risk of pneumonia (lung infection), muscle weakness and problems with swallowing and the voicebox (larynx).

A new treatment called extracorporeal carbon dioxide removal (ECCO2R), has recently been approved by the regulators and is in use at St Thomas’ Hospital. This treatment may allow patients with COPD who are becoming sicker on NIV to avoid going on to a ventilator. ECCO2R involves a cannula (or tube) being placed into either the neck or groin to remove blood from the body. The blood is continuously passed through a machine and the CO2 is removed before it is returned to the blood stream. It is possible for the patient to remain awake, eat and drink and even get out of bed during this treatment. Although we know that the CO2 levels can be reduced in the blood stream using this machine, we do not know what effects it has on the symptoms of breathlessness, on the patient’s ability to breath or how acceptable this treatment is to patients compared with NIV. We also do not know if this treatment improves the chance of survival of patients who are failing NIV.

This study aims to provide information to start answering these questions. We would like to see whether adding ECCO2R to NIV in patients who are at high risk of failing NIV can help prevent the need for a mechanical ventilator, whether this can help them survive and what effects this has on the patient’s symptoms and breathing. We also want to assess how comfortable this approach is compared with the tight fitting mask used for NIV. This study is also being undertaken for educational purposes, as part of a PhD degree at King’s College London.

To assess the breathing we will use a number of tests that are in common use already. All require the patient to breathe and do not cause pain. The tests include:

- Electrical Impedance Tomography (EIT) gives a picture of the lungs during breathing. It involves placing a band of sensors around the chest (similar to the way the heart rate is monitored), and sending a tiny electrical current between these sensors. Aside from the sensation of the band (similar tightness to a belt), there is no discomfort associated with the test. The information from these sensors allows a moving image of your breathing to be shown on a computer screen. The medical team can then use these images to see where the air is within the lungs. This test takes approximately 10-20 minutes.
- Parasternal electromyogram (P-EMG) measures the activity of the muscles in the chest. It also involves placing sensors on the chest and measuring the muscle activity during breathing. This allows us to measure how much effort is being used for each breath. It causes no discomfort. This test takes approximately 10-20 minutes.
- Spirometry is used by medical teams to measure the lung volumes and air movement through the lungs. Patients with COPD have usually had this performed previously by their GP. The test requires breathing out. The test can be used through the NIV machine so that patients do not need to taken off the machine helping them breathe. This test takes approximately 5 minutes.
- Forced oscillometry measures how hard breathing is because of how much wheeze there is. This machine requires them to breathe and causes no discomfort. This test takes approximately 10-20 minutes.
- Oesophageal manometry is a test where we measure the pressure inside the stomach and oesophagus (food pipe connecting the mouth to the stomach). This is performed through a feeding tube that is placed into the stomach from the nose. The test will only be performed if the doctors looking after him/her feel that a feeding tube is required and he/she agrees to having the feeding tube placed. The measurements cause no discomfort and measure the pressures as he/she breathe. This test takes approximately 5-10 minutes.

**2. Why has my relative been chosen?**

Your relative has been chosen to take part because they have been admitted to hospital with an exacerbation of COPD and have acid and CO2 levels in their blood that make it more likely that he/she will need to go onto NIV/ ventilator.

**3. Does my relative have to take part?**

No. Participation is entirely voluntary, and it is up to you to decide whether or not you think that he/she want to take part. If you do decide that they would want to take part, you will be given this information sheet to keep and you will be asked to give permission by signing a consultee declaration form. You are still free to withdraw them from the study at any time and without giving a reason. Once they regain the capacity to decide for themselves, they will be given information about the study (verbal and written) and can decide whether or not they wish to remain in the study. They are free to withdraw from the study at any time and without giving a reason. If you decide that they would not want to take part, or you/they decide to withdraw at any time, their care will not be affected in any way.

**4. What will happen to me if I take part?**

Your relative/friend will be randomly allocated to being started on ECCO2R in addition to NIV or kept on NIV alone. If your relative/friend is allocated to NIV alone then they will remain on NIV for as long as they need it. If your relative/friend is allocated to having ECCO2R added to NIV, then they remain on NIV but will also have a cannula inserted into either their neck or their groin. The cannula will be placed by experienced intensive care doctors using local anaesthetic to make the procedure more comfortable. The cannula takes about 10-20 minutes to place. Once the cannula is in place, the machine is connected and will draw some blood from their body, remove the CO2 and then return it to their body. Your relative/friend will be started on a blood thinning drug to prevent clots forming in the machine. The NIV and ECCO2R will remain for as long as they need them to. Once these are done, we will perform the measurements listed above. All measurements ask them to breathe and each test takes 5-20 minutes to perform depending on the test. The researcher will speak to them before any test is performed and if they do not want to complete any of the tests at any time during the study they do not have to. These tests will happen at the start, after 6-12 hours and then daily. We will ask him/her for their opinion of the comfort of the NIV mask and the cannula and ask questions about their quality of life as it relates to their health. A number of other tests of the blood and urine will occur during the stay in intensive care – almost all of these are part of their normal care in intensive care. We will perform one additional blood test each day for which we need a small amount of blood (no more than 5 mL). Once your relative leaves hospital then we will phone 3 months later and ask some questions about their breathing and general health (about 20-30 minutes in total).

**5. What does my relative have to do?**

For the insertion of the cannula they can decide whether it goes into the neck (in which case we need to lie them down flat and briefly cover their face to keep the cannula clean) or the groin (this needs them to lie with a straight hip but not lie flat). For the tests, they will just need to breathe. For the questions about comfort on the different machines he/she will need to indicate how comfortable they are. When we phone after the study they will need to answer some simple questions about level of activity and breathlessness.

**6. What are the possible disadvantages, precautions or risks of taking part?**

The risks are associated with insertion of the cannula and running the ECCO2R machine. ECCO2R is in routine clinical use at St Thomas’ and is managed by a team of experienced doctors and nurses. The risks of serious or life threatening problems occurring with the cannula insertion is very low (less than 1 in 100). The cannulae are put in using ultrasound guidance to reduce the risk of problems. The common problem is discomfort at the insertion site. Serious and life threatening risks are very rare but are known to include damage to the vein or artery, misplacement of the cannula, abnormal heart rhythm and bleeding or infection at the cannula site. In the experience of the doctors at St Thomas’ the risk of any of these occurring is less than 1 in 100. The cannula is similar to other tubes that are placed in the neck or groin veins during an exacerbation of emphysema and carries the same level of risk.

The potential complications whilst ECCO2R is running are very rare and include clot formation within the device or the blood vessel (reduced by anticoagulation), air entrainment into the device (reduced by safety mechanisms within the device), transient low body temperature, breakdown of the red blood cells, called haemolysis and bleeding. Bleeding can occur in any patient placed on blood thinning agents and although rare (less than 2%) can be significant, requiring blood transfusion and potentially lead to life threatening bleeding (into the brain or internally). Bleeding risk is reduced by regularly measuring the effect of blood thinning agents on the ability of the blood to clot.

There are no risks with the test measurements.

**7. What are the possible benefits of taking part?**

The potential benefit may be from the additional detailed lung function measurements that may allow further optimisation of NIV. It is possible that some people may find ECCO2R more acceptable/comfortable than NIV. All management will occur in a nationally recognised severe respiratory failure centre with expertise in ECCO2R and NIV. All information will be shared with the treating clinician and further management will be left at their discretion.

**8. What happens when the research study stops?**

Once the study is complete, he/she will continue to receive whatever supportive care is required. If the study is stopped for any reason, you and your relative will be told why and continuing care will be arranged. A decision to stop the study could be made by the Sponsor, the Regulatory Authorities, the study researcher, or the Ethics Committee that has approved and is overseeing the study.

**9. What if there is a problem?**

Any possible harm he/she might suffer or any complaints about the way you have been dealt with during the study will be addressed. This is explained in Part 2.

**10. Will my taking part in this study be kept confidential?**

Yes. All the information about participation in this study will be kept confidential. This is explained in Part 2.

**11. Contact Details**

Your study researcher, Dr Nicholas Barrett (Tel: 020 7188 7188, ext. 83038) or his/her delegate will answer any questions you may have.

## If you have additional questions or concerns regarding research, you can contact the Patient Advice and Liaison Service (PALS) at Guy’s and St Thomas’ NHS Foundation Trust on 020 7188 8801.

**This completes Part 1 of the Information Sheet.**

**If the information in Part 1 has interested you and you are considering your relative’s participating, please continue to read the additional information in Part 2 before making any decision.**

**PART 2**

**12. What if relevant new information becomes available?**

Sometimes during the course of a research study, new information becomes available about the question being studied. You and your relative will be informed of any new available information made available. If you or he/she decide not to carry on, the doctor will ensure that all care is continued. If you/he/she decide to continue in the study, you will be asked to sign an updated consultee declaration form on behalf of your relative.

Also, on receiving new information, the study researcher might consider it to be in your relative’s best interest to withdraw him/her from the study. He/she will explain the reasons and arrange for care to continue.

**13. What will happen to my friend/relative if they do not wish to remain in the study?**

You and your relative are free to withdraw from the study at any time and without giving a reason. At any time, a decision to withdraw will not affect the care he/she receives.

If he/she does withdraw during the study, we would like to use the data collected up to the point of your withdrawal. However, he/she/you have the right to withdraw all data related to participation in the study.

**14. What if there is a problem?**

If you or your friend/relative have a concern about any aspect of this study, you or they should speak with the study researcher who will do their best to answer any questions (Tel: 020 7188 7188, ext. 83038). If you or your friend/relative remain unhappy and wish to complain formally, you can do this through the Guy’s and St Thomas’ NHS Foundation Trust complaints procedure. Further details can be found at <http://www.guysandstthomas.nhs.uk/patients-and-visitors/patients/raising-concerns.aspx> or through the Patient Advice and Liaison Service (PALS) at Guy’s and St Thomas’ NHS Foundation Trust.

If your friend/relative receives a physical injury as a direct result of being in this study, the medical team will treat your friend/relative accordingly or refer your friend/relative for treatment. This treatment will be provided at no cost to your friend/relative . All professional staff involved in the study hold professional indemnity to work within Guy’s and St Thomas’ NHS Trust.  In the event that they are harmed during the research and this is due to negligence then they may have grounds for legal action for compensation against Guy’s and St Thomas’ NHS Trust but they may have to pay their legal costs.  The normal NHS complaints mechanisms are still available.

The sponsors will at all times maintain adequate insurance in relation to the study independently. Guy’s and St Thomas’ NHS Foundation Trust has a duty of care to patients via NHS indemnity cover in respect of any claims arising as a result of clinical negligence by its employees, brought by or on behalf of a study patient.

**15. Will my relative/friend’s taking part in this study be kept confidential?**

The handling of medical information obtained in clinical research is controlled by national and international data protection regulations and medical confidentiality. Your relative has the right to control the use of their medical information, to ask for updated information on what data are recorded and to request the correction of errors.

Study data will be made anonymous by assigning a unique number to them. All data collected from them will then be identified by this number. No data which could be used to identify him/her will be transferred from the medical notes.

The medical information collected during this study will first be checked to make sure it is true and accurate. It will then be transferred into study database(s) and processed to allow the results of this study to be analysed and reported or published for scientific purposes.

Your relative’s identity will be kept confidential at all times, except to those professionals who need to check study data. If he/she does participate in the study, the medical records and the data collected for the study may be looked at by authorised persons from the sponsor organising the research, auditors, institutional review boards, and independent ethics committees or by any other regulatory bodies. All the people who may look at the data have a duty of confidentiality to your relative, as a research participant, and nothing that could reveal his/her identity will be disclosed by these persons outside of the hospital. By signing the consent form, you are authorising these professional groups access to your relative’s data and medical notes, if necessary.

**16. What will happen to the results of the research study?**

The results of the study will be submitted to one or more sponsor offices or regulatory authorities, and may also be published. The data collected from your participation in this study are considered personal data as defined under European Union Directive 95/46/EC. The data controller at the sponsor institution will take steps to ensure that personal data are protected. Consent is needed for the data to be used for these purposes. Your relative will not be identified personally in any publication resulting from the study.

**17. Who is organising and funding the research?**

The research is being organised by members of the Intensive Care Department at Guy’s & St Thomas’ NHS Foundation Trust. The equipment and salary for one of the doctors (research fellow) is being funded by ALung Incorporated.

**18. Who has reviewed the study?**

This study has been reviewed by the East of England - Cambridge Central Research Ethics Committee, the London Respiratory Physiology Research Group and the King’s Health Partners Critical Care and Peri-Operative Research Group.

**THANK YOU FOR CONSIDERING WHETHER YOUR RELATIVE/FRIEND SHOULD TAKE PART IN THIS STUDY.**
