# Supplementary material for: Extracorporeal carbon dioxide removal for acute hypercapnic exacerbations of chronic obstructive pulmonary disease: study protocol for a randomised controlled trial
Source: Trials. 2019 Jul 30;20:465. doi: 10.1186/s13063-019-3548-4 (PMC6664508; doi:10.1186/s13063-019-3548-4)
Supplement: Supplementary file 1 — Contains a consultee assent form, Consultee information sheet, the participant consent and the participant information sheet. (ZIP 339 kb) [file 13063_2019_3548_MOESM1_ESM.zip › Participant Consent formR1.docx]

**Participant Study Number:**

**PARTICIPANT CONSENT FORM**

**Project title: Extra-corporeal CO_2_ Removal as an adjunct to Non-Invasive Ventilation in Acute Severe Exacerbations of COPD**

**Ethics Reference:** 14/EE/0109

**Research & Development Reference:**

**Name of Principle Investigator:** Dr Nicholas Barrett

**Please initial box**

| I confirm that I have read and understood the ‘Participant Information Sheet’ (Version 3.1, dated 16/1/2016) for the above study and have had the opportunity to ask questions and have had these answered satisfactorily. | ⬜ |
| --- | --- |
| I understand that my participation is voluntary and that I am free to withdraw at any time, without giving any reason, and without my medical care or legal rights being affected. | ⬜ |
| I understand that relevant sections of my medical notes and data collected during the study may be looked at by responsible individuals from Guy’s & St Thomas’ NHS Foundation Trust or from regulatory authorities, where appropriate and relevant to my taking part in the above research. I give permission for these individuals to have access to my records. | ⬜ |

| I give permission for my GP to be informed of my enrolment in the above study. | ⬜ |
| --- | --- |

| I agree to take part in the above study. | ⬜ |
| --- | --- |

________________________ ________________ ____________________

Name of Patient Date Signature

_________________________ ________________ ____________________

Name of Study Researcher Date Signature

When completed: 1 (original) to be kept in care record, 1 for patient; 1 for researcher site file
