# Supplementary material for: Extracorporeal carbon dioxide removal for acute hypercapnic exacerbations of chronic obstructive pulmonary disease: study protocol for a randomised controlled trial
Source: Trials. 2019 Jul 30;20:465. doi: 10.1186/s13063-019-3548-4 (PMC6664508; doi:10.1186/s13063-019-3548-4)
Supplement: Supplementary file 1 — Contains a consultee assent form, Consultee information sheet, the participant consent and the participant information sheet. (ZIP 339 kb) [file 13063_2019_3548_MOESM1_ESM.zip › Participant information sheetR1.docx]

**Participant Information Sheet**

**Principal Investigator:** Dr Nicholas Barrett

**Location/Study Site:** St Thomas’ Hospital, London, SE1 7EH

**Sponsor:** Guy’s & St. Thomas’ NHS Foundation Trust.

**Participant Study Number:**

**Extra-corporeal CO_2_ Removal as an adjunct to Non-Invasive Ventilation
in Acute Severe Exacerbations of COPD**

**Ethics Reference:** 14/EE/0109

**Research & Development Reference:**

**Introduction:**

We would like to invite you to take part in our research study. Before you decide, we would like you to understand why the research is being done and what it would involve for you. Please take time to read this document carefully and ask any questions you may have about the information below or any words you may not understand. You may discuss it with others if you wish

If you decide that you wish to take part in our research study, you will be asked to read and sign a consent form. You will then be given a copy to keep.

If you do not wish to participate in our research study or you wish to withdraw from the study after consenting, please say so, as your participation is entirely voluntary. This will not affect the standard of clinical care that you receive.

**Part 1** tells you the purpose of the study and what will happen to you if you take part.

**Part 2** gives you more detailed information about the conduct of the study.

Please ask the study researcher if there is anything that is not clear or if you would like more information. Take time to decide whether or not you wish to take part.

**PART 1:**

**1. What is the purpose and background of the study?**

Patients with long-standing chronic obstructive pulmonary disease (COPD) or emphysema commonly have acute exacerbations of their disease causing increased breathlessness, cough and phlegm (or sputum). When exacerbations are mild, they are treated with medications prescribed by GPs and the patient can usually stay at home. More severe exacerbations usually require hospital admission. When exacerbations are very severe and breathing is very difficult, then the carbon dioxide (CO2) levels in the blood can rise. If the CO2 level becomes high enough, the acid levels in the blood rise and the patient can become progressively distressed. If a patient is this ill, the usual treatment often includes prednisolone (a steroid tablet), nebulisers and antibiotics. If the CO2 or acid levels in the blood remain high then patients are usually started on a breathing machine called non-invasive ventilation (NIV), which improves breathing by giving extra pressure to reduce the effort of each breath. Research studies in the UK and overseas have previously shown that using NIV for exacerbations of COPD can improve survival rate. NIV is given through a mask fitted tightly onto the patient’s face. The mask needs to be tight to prevent air-leak and facilitate the correct functioning of the breathing machine. Patients who are started on NIV have a good chance of surviving to leave hospital (approximately 14 out of 15 patients). However some patients, approximately 1 in 4 or 5, fail NIV. Some patients fail NIV because they find it uncomfortable, whilst other patients get sicker despite NIV. If a patient fails NIV, usually the next level of treatment is for the doctors to insert a breathing tube through the mouth down into the trachea (the large airway leading air from the mouth to the lungs) under deep sedation and to start on a different kind of breathing machine called a mechanical ventilator. Approximately 1 in 3 patients with COPD who are started on a mechanical ventilator do not survive, because of increased risk of pneumonia (lung infection), muscle weakness and problems with swallowing and the voicebox (larynx).

A new treatment called extracorporeal carbon dioxide removal (ECCO2R), has recently been approved by the regulators and is in use at St Thomas’ Hospital. This treatment may allow patients with COPD who are becoming sicker on NIV to avoid going on to a ventilator. ECCO2R involves a cannula (or tube) being placed into either the neck or groin to remove blood from the body. The blood is continuously passed through a machine and the CO2 is removed before it is returned to the blood stream. It is possible for the patient to remain awake, eat and drink and even get out of bed during this treatment. Although we know that the CO2 levels can be reduced in the blood stream using this machine, we do not know what effects it has on the symptoms of breathlessness, on the patient’s ability to breath or how acceptable this treatment is to patients compared with NIV. We also do not know if this treatment improves the chance of survival of patients who are failing NIV.

This study aims to provide information to start answering these questions. We would like to see whether adding ECCO2R to NIV in patients who are at high risk of failing NIV can help prevent the need for a mechanical ventilator, whether this can help them survive and what effects this has on the patient’s symptoms and breathing. We also want to assess how comfortable this approach is compared with the tight fitting mask used for NIV. This study is also being undertaken for educational purposes, as part of a PhD degree at King’s College London.

To assess the breathing we will use a number of tests that are in common use already. All require the patient to breathe and do not cause pain. The tests include:

- Electrical Impedance Tomography (EIT) gives a picture of the lungs during breathing. It involves placing a band of sensors around your chest (similar to the way your heart rate is monitored), and sending a tiny electrical current between these sensors. Aside from the sensation of the band (similar tightness to a belt), there is no discomfort associated with the test. The information from these sensors allows a moving image of your breathing to be shown on a computer screen. The medical team can then use these images to see where the air is within the lungs. This test takes approximately 10-20 minutes.
- Parasternal electromyogram (P-EMG) measures the activity of the muscles in the chest. It also involves placing sensors on your chest and measuring the muscle activity as you breathe. This allows us to measure how much effort you are using for each breath. It causes no discomfort. This test takes approximately 10-20 minutes.
- Spirometry is used by medical teams to measure the lung volumes and air movement through your lungs. Patients with COPD have usually had this performed previously by their GP. The test requires you breathe out. The test can be used through the NIV machine so that patients do not need to taken off the machine helping them breathe. This test takes approximately 5 minutes.
- Forced oscillometry measures how hard it is for you to breathe because of how wheezy you are. This machine requires you to breathe and causes no discomfort. This test takes approximately 10-20 minutes.
- Oesophageal manometry is a test where we measure the pressure inside your stomach and oesophagus (food pipe connecting the mouth to the stomach). This is performed through a feeding tube that is placed into your stomach from your nose. The test will only be performed if the doctors looking after you feel that a feeding tube is required and you agree to having the feeding tube placed. The measurements cause no discomfort and measure the pressures as you breathe. This test takes approximately 5-10 minutes.

**2. Why have I been chosen?**

You have been chosen to take part because you have been admitted to hospital with an exacerbation of COPD and have acid and CO2 levels in your blood that make it more likely that you will need to go onto a NIV/ventilator.

**3. Do I have to take part?**

No. Your participation is entirely voluntary, and it is up to you to decide whether or not you want to take part. If you do decide that you want to take part, you will be given this information sheet to keep and you will be asked to give permission by signing a consent form. You are still free to withdraw yourself from the study at any time and without giving a reason. If you decide that you do not want to take part, or you decide to withdraw at any time, your care will not be affected in any way.

**4. What will happen to me if I take part?**

You will be randomly allocated to being started on ECCO2R in addition to NIV or kept on NIV alone. If you are allocated to having ECCO2R added to NIV, then you remain on NIV but will also will have a cannula inserted into either your neck or your groin. The cannula will be placed by experienced intensive care doctors using local anaesthetic to make the procedure more comfortable. The cannula takes about 10-20 minutes to place. Once the cannula is in place, the machine is connected and will draw some blood from your body, remove the CO2 and then return it to your body. You will be started on a blood thinning drug to prevent clots forming in the machine. The NIV and ECCO2R will remain for as long as you need them to. If you are allocated to NIV alone then you will remain on NIV for as long as you need it. Once these are done, we will perform the measurements listed above. All measurements ask you to breathe and each test takes 5-20 minutes to perform depending on the test. The researcher will speak to you before any test is performed and if you do not want to complete any of the tests at any time during the study you do not have to. These tests will happen at the start, after 6-12 hours and then daily. We will ask you for your opinion of the comfort of the NIV mask and the cannula and ask questions about your quality of life as it relates to your health. A number of other tests of your blood and urine will occur during your stay in intensive care – almost all of these are part of your normal care in intensive care. We will perform one additional blood test each day for which we need a small amount of blood (no more than 5 mL). Once you leave hospital then we will call you 3 months later and ask you some questions about your breathing and general health (about 20-30 minutes in total).

**5. What do I have to do?**

For the insertion of the cannula you can decide whether it goes into the neck (in which case we need to lie you down flat and briefly cover your face to keep the cannula clean) of the groin (this needs you to lie with a straight hip but not lie flat). For the tests, you will just need to breathe. For the questions about your comfort on the different machines you will need to indicate how comfortable you are. When we call you after the study you will need to answer some simple questions about your level of activity and breathlessness.

**6. What are the possible disadvantages, precautions or risks of taking part?**

The risks are associated with insertion of the cannula and running the ECCO2R machine. ECCO2R is in routine clinical use at St Thomas’ and is managed by a team of experienced doctors and nurses. The risks of serious or life threatening problems occurring with the cannula insertion is very low (less than 1 in 100). The cannulae are put in using ultrasound guidance to reduce the risk of problems. The common problem is discomfort at the insertion site. Serious and life threatening risks are very rare but are known to include damage to the vein or artery, misplacement of the cannula, abnormal heart rhythm and bleeding or infection at the cannula site. In the experience of the doctors at St Thomas’ the risk of any of these occurring is less than 1 in 100. The cannula is similar to other tubes that are placed in the neck or groin veins during an exacerbation of emphysema and carries the same level of risk.

The potential complications whilst ECCO2R is running are very rare and include clot formation within the device or the blood vessel (reduced by anticoagulation), air entrainment into the device (reduced by safety mechanisms within the device), transient low body temperature, breakdown of the red blood cells, called haemolysis and bleeding. Bleeding can occur in any patient placed on blood thinning agents and although rare (less than 2%) can be significant, requiring blood transfusion and potentially lead to life threatening bleeding (into the brain or internally). Bleeding risk is reduced by regularly measuring the effect of blood thinning agents on the ability of the blood to clot.

There are no risks with the test measurements.

**7. What are the possible benefits of taking part?**

The potential benefit for you may be from the additional detailed lung function measurements that may allow further optimisation of NIV. It is possible that some people may find ECCO2R more acceptable/comfortable than NIV. All of your management will occur in a nationally recognised severe respiratory failure centre with expertise in ECCO2R and NIV. This information will be shared with the treating clinician and further management will be left at their discretion.

**8. What happens when the research study stops?**

Once the study is complete, you will continue to receive whatever supportive care you require. If the study is stopped for any reason, you will be told why and your continuing care will be arranged. A decision to stop the study could be made by the Sponsor, the Regulatory Authorities, your study researcher, or the Ethics Committee that has approved and is overseeing the study.

**9. What if there is a problem?**

Any possible harm you might suffer or any complaints about the way you have been dealt with during the study will be addressed. This is explained in Part 2.

**10. Will my taking part in this study be kept confidential?**

Yes. All the information about your participation in this study will be kept confidential. This is explained in Part 2.

**11. Contact Details**

Your study researcher, Dr Nicholas Barrett (Tel: 020 7188 7188, ext. 83038) or his/her delegate will answer any questions you may have.

## If you have additional questions or concerns regarding research, you can contact the Patient Advice and Liaison Service (PALS) at Guy’s and St Thomas’ NHS Foundation Trust on 020 7188 8801.

**This completes Part 1 of the Information Sheet.**

**If the information in Part 1 has interested you and you are considering participating, please continue to read the additional information in Part 2 before making any decision. PART 2**

**12. What if relevant new information becomes available?**

Sometimes during the course of a research study, new information becomes available about the question being studied. You will be informed of any new available information made available. If you decide not to carry on, your doctor will ensure that your care is continued. If you decide to continue in the study, you will be asked to sign an updated consent form.

Also, on receiving new information, your study researcher might consider it to be in your best interest to withdraw you from the study. He/she will explain the reasons and arrange for your care to continue.

**13. What will happen if I don’t want to carry on with the study?**

You are free to withdraw from the study at any time and without giving a reason. At any time, a decision to withdraw will not affect the care you receive.

If you withdraw during the study, we would like to use the data collected up to the point of your withdrawal. However, you have the right to withdraw all data related to your participation in the study.

**14. What if there is a problem?**

If you have a concern about any aspect of this study, you should speak with your study researcher who will do their best to answer your questions (Tel: 020 7188 7188, ext. 83038). If you remain unhappy and wish to complain formally, you can do this through the Guy’s and St Thomas’ NHS Foundation Trust complaints procedure. Further details can be found at <http://www.guysandstthomas.nhs.uk/patients-and-visitors/patients/raising-concerns.aspx> or through the Patient Advice and Liaison Service (PALS) at Guy’s and St Thomas’ NHS Foundation Trust.

If you receive a physical injury as a direct result of being in this study, the intensive care medical team will treat you accordingly or refer you for treatment. This treatment will be provided at no cost to you. All professional staff involved in the study hold professional indemnity to work within Guy’s and St Thomas’ NHS Trust.  In the event that you are harmed during the research and this is due to negligence then you may have grounds for legal action for compensation against Guy’s and St Thomas’ NHS Trust but you may have to pay your legal costs.  The normal NHS complaints mechanisms are still available to you.

The sponsors will at all times maintain adequate insurance in relation to the study independently. Guy’s and St Thomas’ NHS Foundation Trust has a duty of care to patients via NHS indemnity cover in respect of any claims arising as a result of clinical negligence by its employees, brought by or on behalf of a study patient.

**15. Will my taking part in this study be kept confidential?**

The handling of medical information obtained in clinical research is controlled by national and international data protection regulations and medical confidentiality. You have the right to control the use of your medical information, to ask for updated information on what data are recorded and to request the correction of errors.

Your study data will be made anonymous by assigning a unique number to you. All data collected from you will then be identified by this number. No data which could be used to identify you will be transferred from your medical notes.

The medical information collected during this study will first be checked to make sure it is true and accurate. It will then be transferred into study database(s) and processed to allow the results of this study to be analysed and reported or published for scientific purposes.

Your identity will be kept confidential at all times, except to those professionals who need to check study data. If you participate in the study, your medical records and the data collected for the study may be looked at by authorised persons from the sponsor organising the research, auditors, institutional review boards, and independent ethics committees or by any other regulatory bodies. All the people who may look at the data have a duty of confidentiality to you, as a research participant, and nothing that could reveal your identity will be disclosed by these persons outside of the hospital. By signing the consent form, you are authorising these professional groups access to your data and medical notes, if necessary.

**16. What will happen to the results of the research study?**

The results of the study will be submitted to one or more sponsor offices or regulatory authorities, and may also be published. The data collected from your participation in this study are considered personal data as defined under European Union Directive 95/46/EC. The data controller at the sponsor institution will take steps to ensure that personal data are protected. Your consent is needed for the data to be used for these purposes. You will not be identified personally in any publication resulting from the study.

**17. Who is organising and funding the research?**

The research is being organised by members of the Intensive Care Department at Guy’s & St Thomas’ NHS Foundation Trust. The equipment and salary for one of the doctors (research fellow) is being funded by ALung Incorporated.

**18. Who has reviewed the study?**

This study has been reviewed by the East of England - Cambridge Central Research Ethics Committee, the London Respiratory Physiology Research Group and the King’s Health Partners Critical Care and Peri-Operative Research Group.

**THANK YOU FOR CONSIDERING TAKING PART IN THIS STUDY.**
